# Supplementary material for: An integrated multi-omics approach to identify regulatory mechanisms in cancer metastatic processes
Source: Genome Biol. 2021 Jan 7;22:19. doi: 10.1186/s13059-020-02213-x (PMC7789593; doi:10.1186/s13059-020-02213-x)
Supplement: Supplementary file 1 — Additional file 1. Supplementary Figures. [file 13059_2020_2213_MOESM1_ESM.pdf]

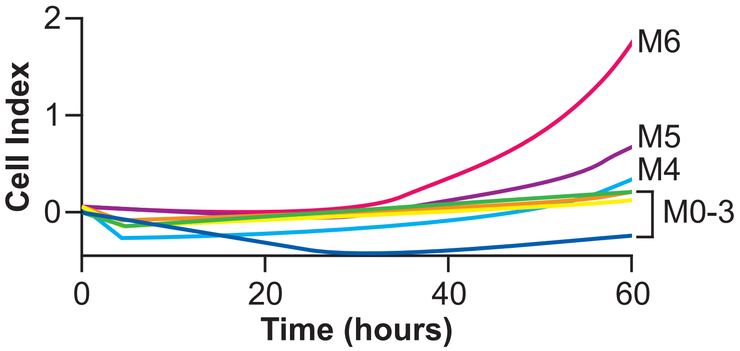

Figure S1. Invasiveness of cell lines after undergoing selection. SW480 cells were subjected to repeated selection for capacity to invade through a Matrigel-coated microporous membrane toward a chemoattractant (fetal bovine serum). The number of times cells were selected using Matrigel-coated membranes is denoted as the “M” number for the cell line. M0, parental SW480 cell line that has not undergone selection. M1-M6, SW480 cells that have been selected 1-6 times using this procedure. Invasive capacity was measured using the xCelligence realtime cell analysis platform with cell invasion migration (CIM) plates. For invasion assessment, invasion chambers were pre-coated with Matrigel prior to plating cells. Fetal bovine serum was used as a chemoattractant. Cell index represent a disruption in the conductance of current at the electrode–media interface on the underside of the microporous membrane. As such increasing cell index corresponds to increasing cell invasion through the membrane. Data were recorded for 60 hours. Each line represents the mean of three independent wells of cells assessed in parallel on the same instrument. All depicted measures were recorded at the same time on the same instrument. M0-3 cell index profiles are largely indistinguishable. M4 cells begin to show invasive capacity, whereas migration is notably higher for M5 and M6 cells.

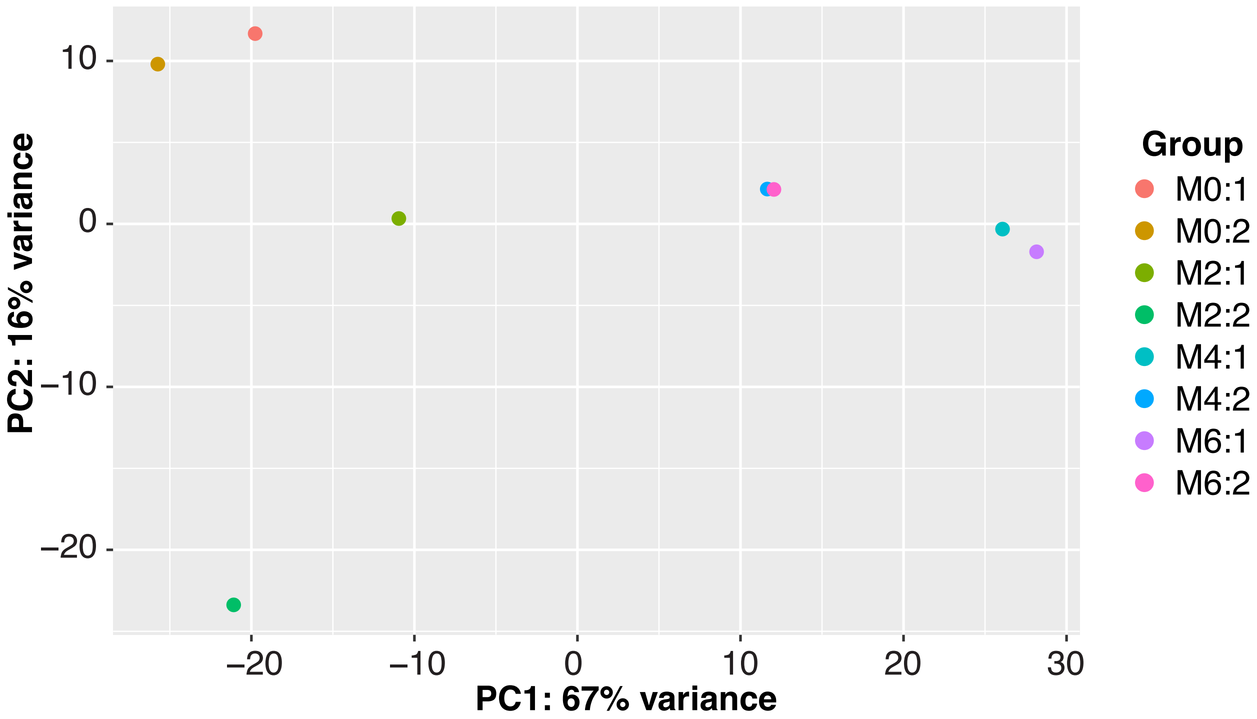

Figure S2. Principal component analysis of SW480-M0–M6 cell lines. Global transcriptome analysis (RNA-seq) was performed on SW480-M0, M2, M4, and M6 cells. Two independently generated cell lines were evaluated for each stage of selection. Principal component analysis was used as an initial assessment of correlation between transcriptome signatures between cell selection stages (e.g., between M0 and M6) as well as between replicate cultures (e.g., between M0:1 and M0:2). Legend: “Group” represents the cell selection stage (i.e., M0–M6) followed by the experimental replicate number (e.g., if the cells were from the first or second replicate experiment to select invasive cells).

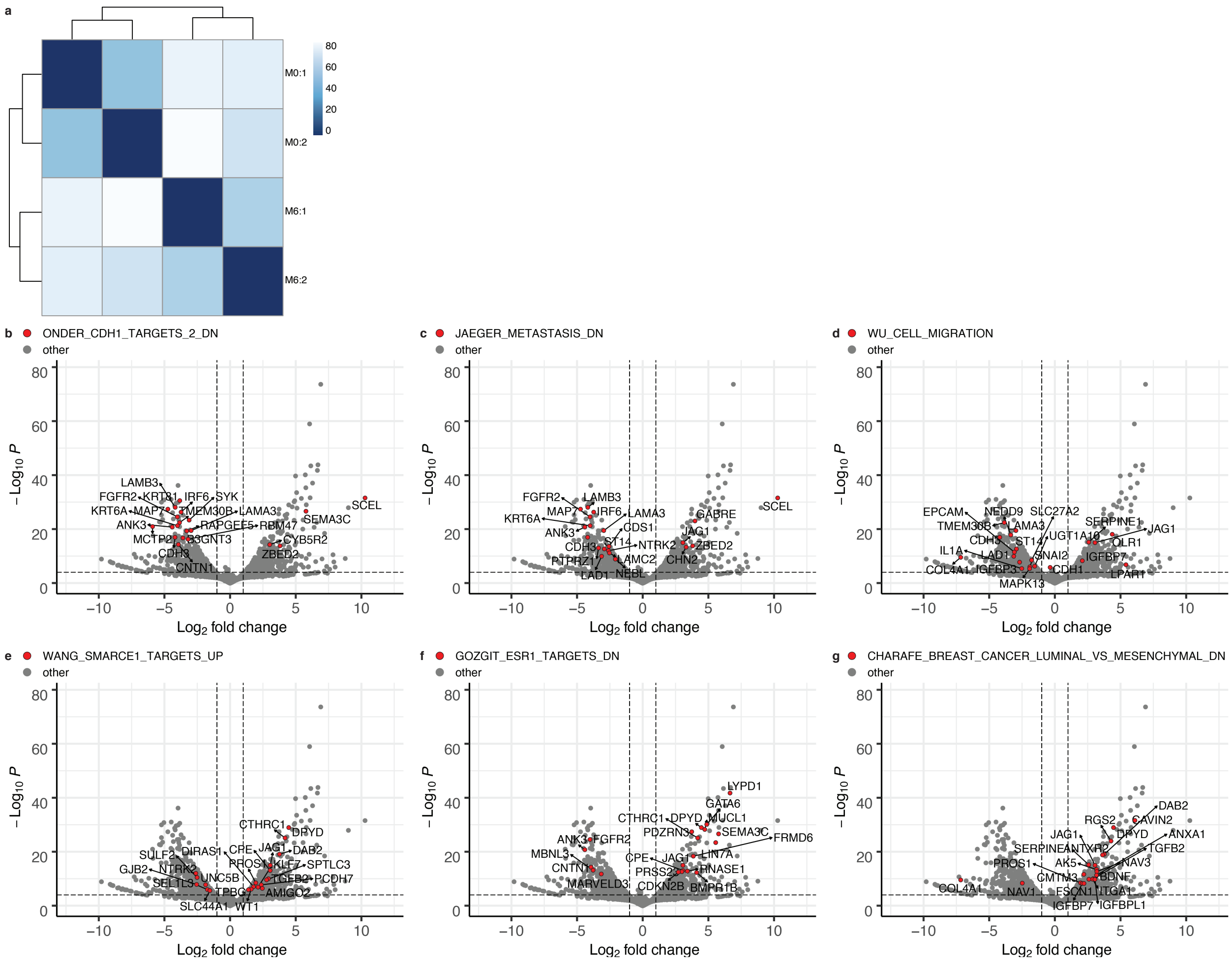

Figure S3. Heatmap and Volcano plot for M0 vs M6. a. To assess similarities and dissimilarities, a distance matrix heatmap was generated using hierarchical clustering of gene expression profiles for two M0 and two M6 cell lines. **b-g.** Volcano plots displaying distributions of p-values by Log2 fold change for strongly associated gene sets differentially expressed between M0 and M6. For each panel the 20 most significant genes within the indicated gene set are denoted with red markers and annotated with gene names.

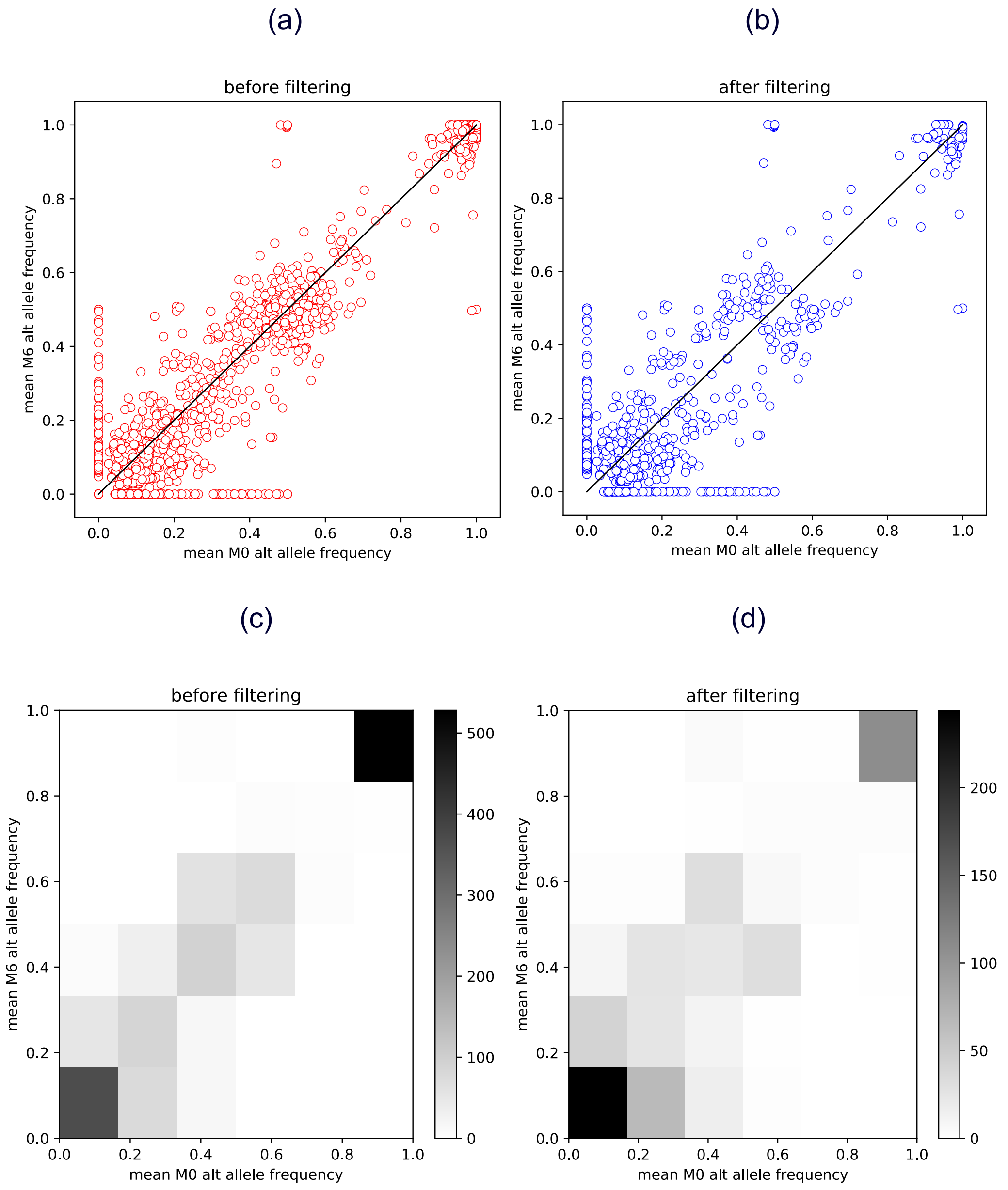

Figure S4. Changes in alternative allele frequencies between early and late stages. Average alternative allele frequency for M6 vs M0 for all the SNVs (n = 1516) having sufficient read depth (average read depth for all SNVs was used as threshold) **(a)**, and the subset of SNVs (n = 674) that exhibit significant change (aggregated binomial test p-value < 0.05) in alternative allele frequency from M0 to M6 **(b)**. The significance of change was determined by testing the deviation of alternative allele frequency for each replicate of M6 stage from the average M0 alternative allele frequency using binomial test and aggregating p-values using Fisher's method. **(c)** and **(d)** are 2D histograms associated with **(a)** and **(b)** respectively.

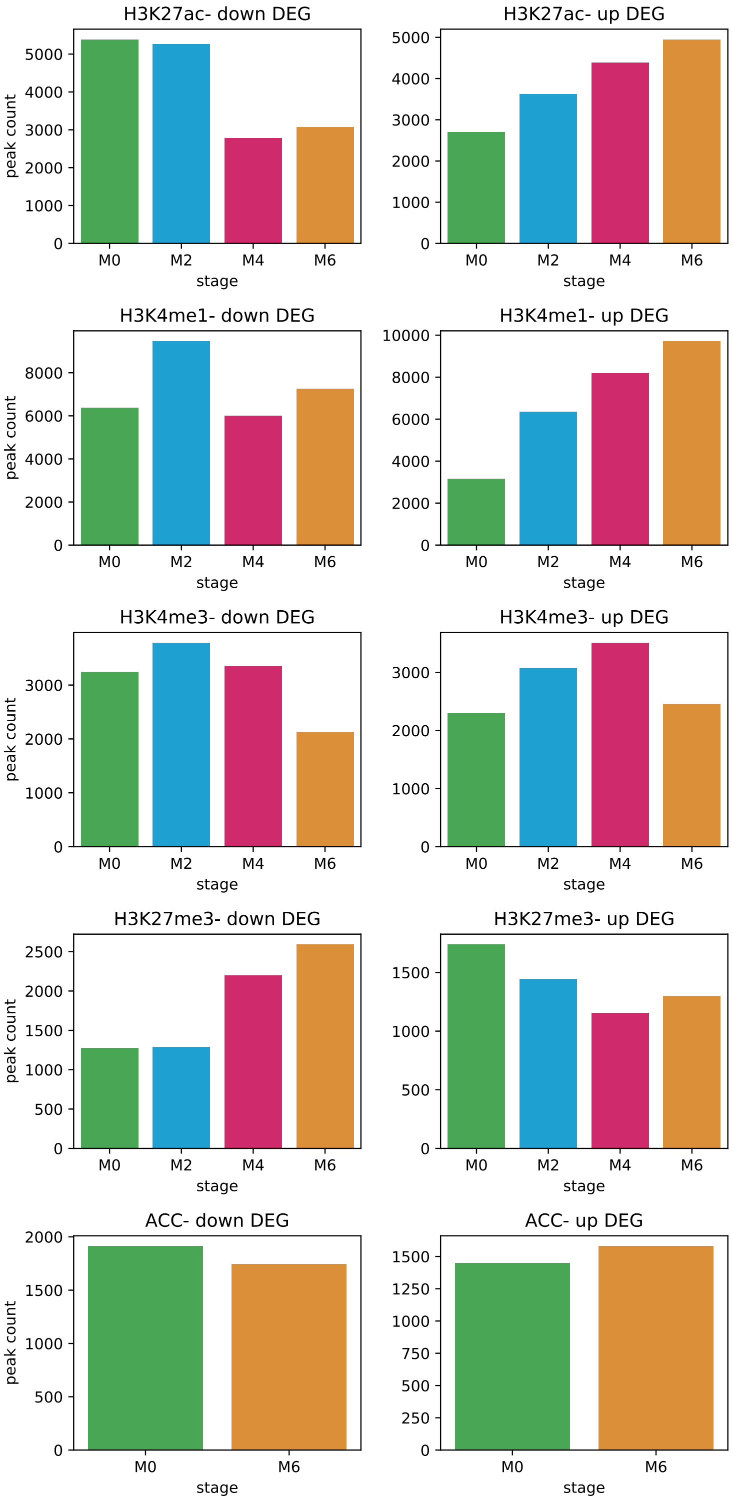

Figure S5. Histone mark and chromatin accessibility changes during progression. For each mark and chromatin accessibility, the count of peaks within 10Kb upstream of the DE genes (DE

p-value < 0.05) is shown for each stage, separately for down-regulated (left) and up-regulated (right) genes. For H3K27ac there is a sharp decrease in the peak count from M0, M2 to M4, M6 and for H3K27me3 there is a sharp increase in the peak count from M0, M2 to M4, M6 near down-regulated genes. There is a gradual increase and a gradual decrease in the peak counts near up-regulated genes for H3K27ac and H3K27me3 respectively. For H3K4me3, there is a monotonic decrease from M2 to M6 and a monotonic increase from M0 to M4 in the peak counts near down and up-regulated genes respectively. Also, comparison of M0 with M6 shows that the peak counts for H3K4me3 increase slightly for up-regulated genes and decrease for down-regulated genes. For H3K4me1 the peak counts monotonically increase near up-regulated genes, whereas near down-regulated genes, the number of peaks goes up for M2 then goes down and there is an increase in the peak count for M6 vs M0. The number of accessible regions does not change substantially between M0 and M6, for either set of DE genes. There is a slight increase and decrease in the number of accessible sites for up- and down-regulated genes respectively. For easier visualization of these minor changes only the count of peaks with height of at least 500 are represented in the plot.

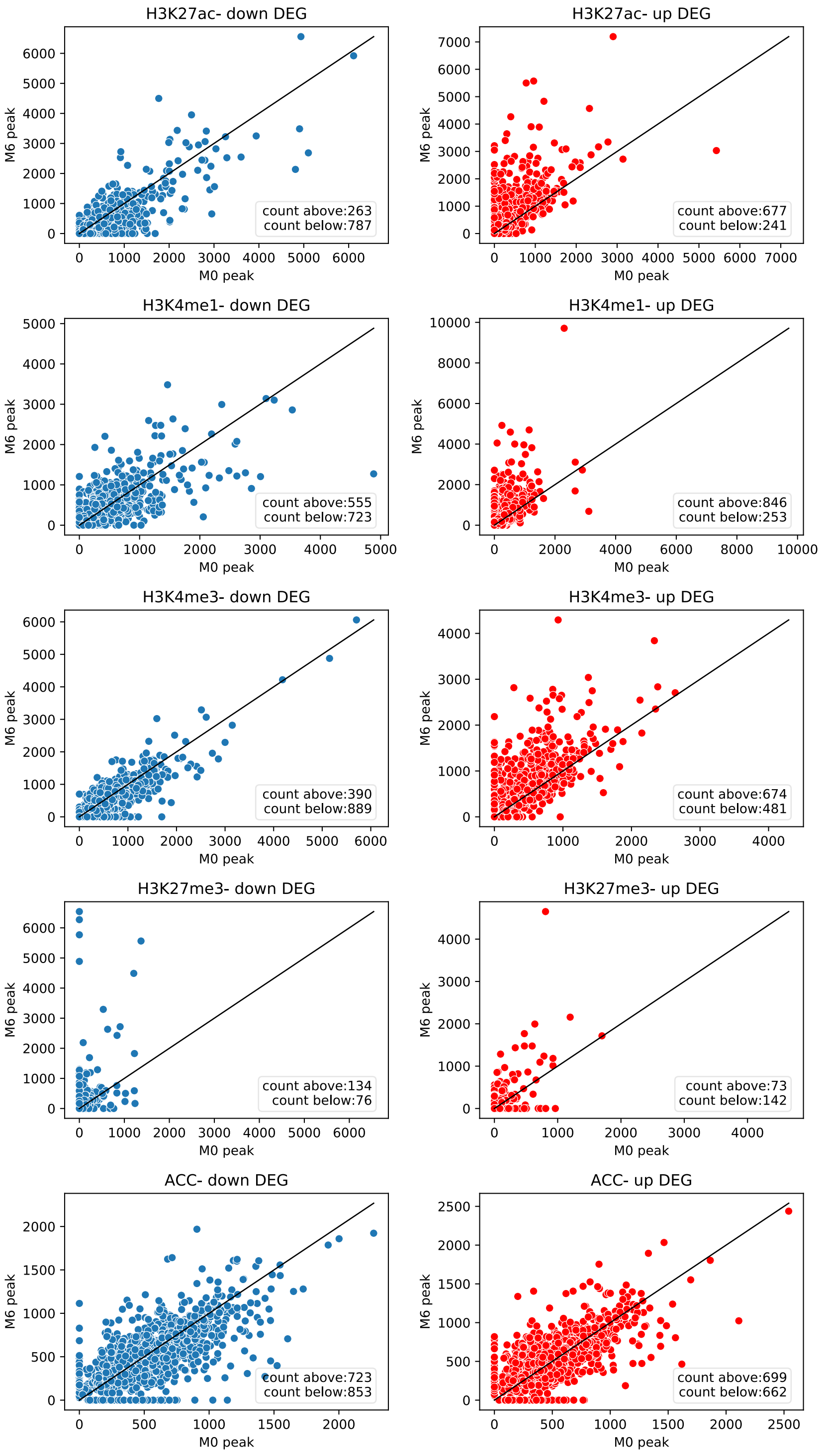

Figure S6. Maximum height of peaks within 10Kb upstream of each DE gene for M6 versus M0. Each plot represents how the maximum height of a histone mark ChIP peak or accessibility (ATAC-seq) peak within 10Kb upstream of each DE gene ("DEG", DE p-value < 0.05) changes in M6 vs M0. The left and right plots show this information for down- and up-regulated genes respectively and the insets show the count of the genes above or below the identity line. For all activating histone marks there is an increase in peak height near the majority of up-regulated

genes whereas there is a decrease in the peak height near the majority of down-regulated genes. The opposite pattern exists for H3K27me3, which is believed to be a repressive mark. For accessibility peaks, there is no significant difference between the number of DE genes on either side of the identity line, suggesting that accessibility strength does not change predominantly in one direction over the other.

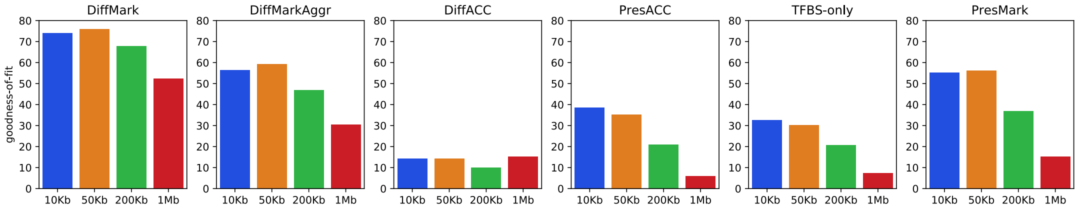

Figure S7. Goodness-of-fit at varying distance thresholds. Comparison of goodness-of-fit for each distance threshold (maximum distance upstream or downstream of gene) used for associating TF ChIP-peaks with genes, shown separately for each strategy. The goodness-of-fit is measured by the sum of test LLRs (derived from cross-validation) in up- and down- analyses, averaged over 100 repeats of the procedure. A distance threshold of 1Mb yields the poorest fits for all strategies except DiffACC.

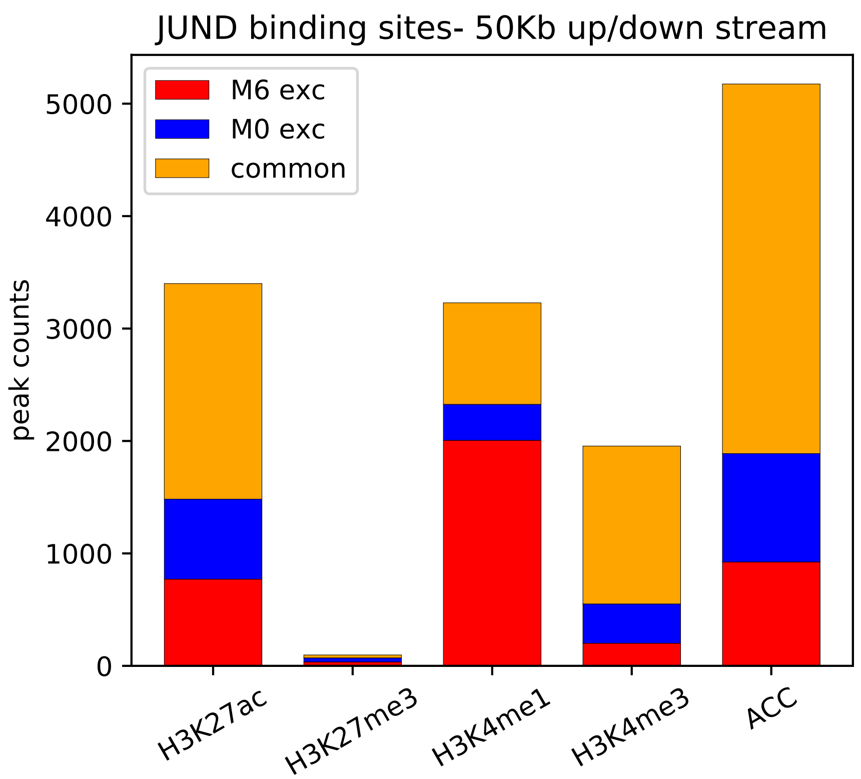

Figure S8. Changes in the number of histone mark and accessibility peaks within JunD binding sites. For each histone mark and for accessibility, the count of the peaks within JunD binding sites, located within 50Kb upstream or downstream of the protein coding genes, is partitioned into three categories. The red bars show the number of peaks exclusive to M6, the blue bars show the number of peaks exclusive to M0, and the yellow bars indicate the number of peaks shared between the two stages. The number of peaks exclusive to M0 is almost equal to the number of exclusive peaks to M6 for H3K27ac and accessibility (ACC). The number of peaks exclusive to M6 is much larger than the number of peaks exclusive to M0 for H3K4me1 and the opposite pattern exists for H3K4me3. For H3K27me3 the total number of peaks is small, and the total number of exclusive peaks is larger than the common peaks. Accessibility and H3K4me1 have the largest and smallest ratio of the number of common sites to the total number of exclusive sites.

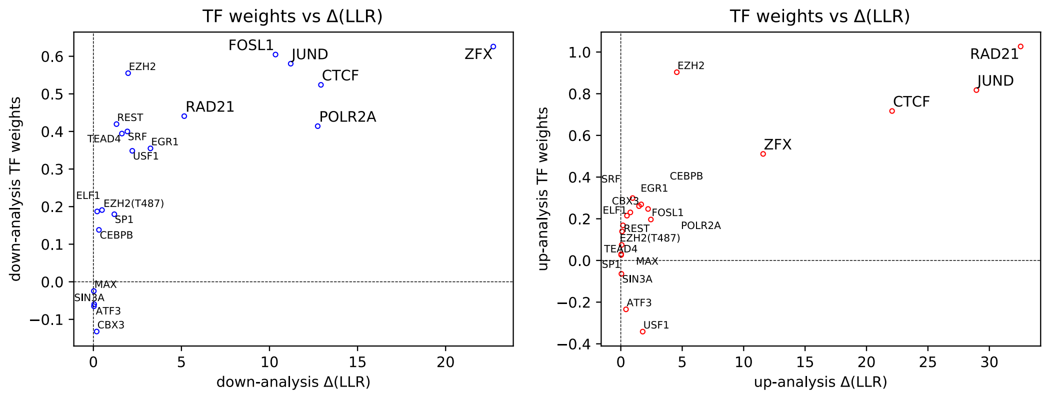

Figure S9. Contributions of individual TFs to the model trained with fw-pGENMi. TF weights learned by fw-pGENMi (y-axis) versus significance of the TF measured by $\Delta(LLR)$ in modeling the data (x-axis), for down-analysis (left) and up-analysis (right). Here the top 6 and top 4 TFs with the highest $\Delta(LLR)$ in down- and up-analysis respectively (larger font size) are the same as the top 6 and top 4 TFs in the TF ranking of the models trained with pGENMi for down- and up-analysis respectively, suggesting that fw-pGENMi with far fewer parameters (28 weight parameters compared) identifies the same top TFs as pGENMi (160 weight parameters).

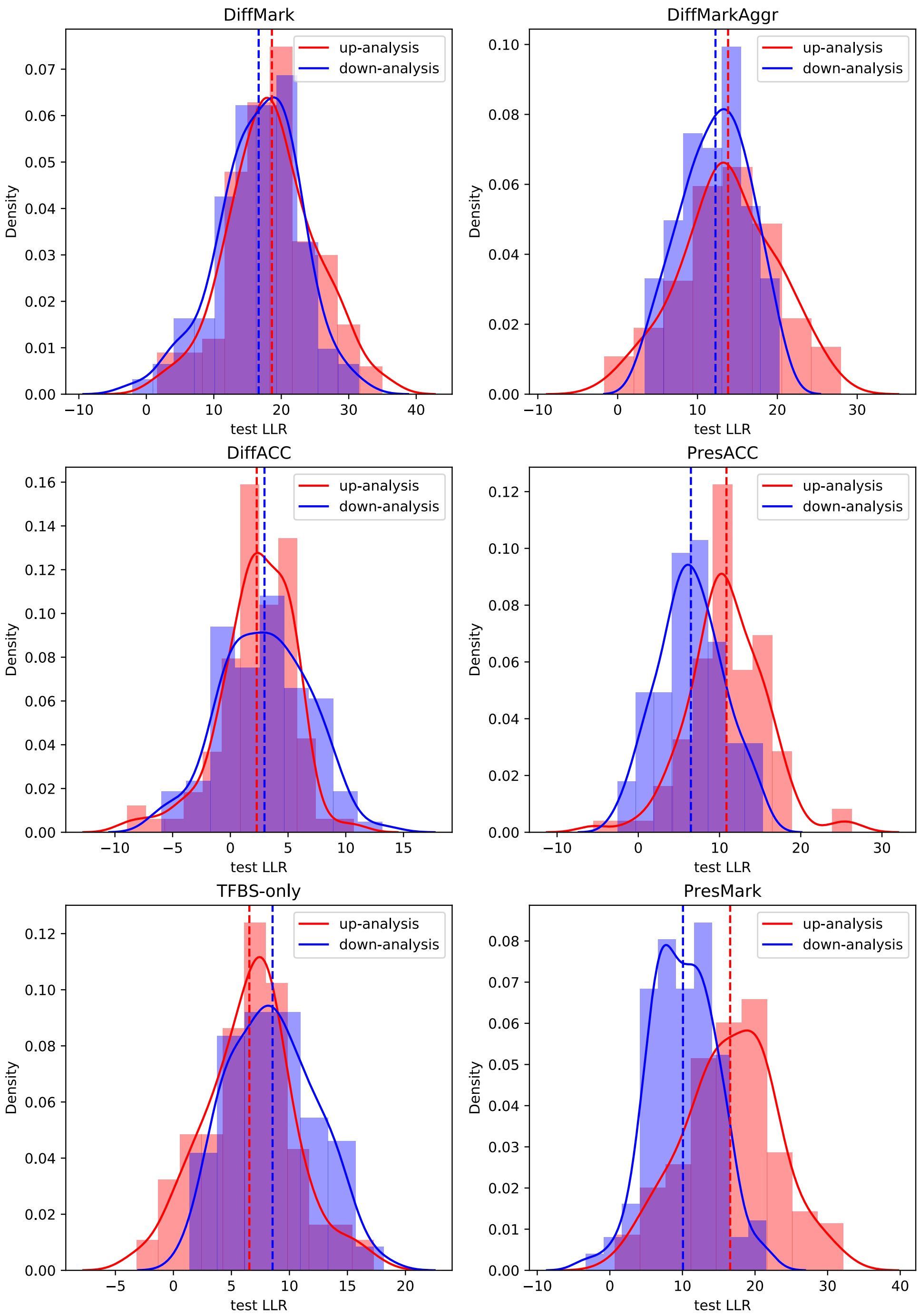

Figure S10. Predictive power under different strategies for defining cis-regulatory evidence. Histogram of test LLRs derived from training-validation-test cross validation for up- and down-analysis in different strategies. Each histogram is based on 100 repetitions of random cross-validation. In both analyses, the average test LLR (shown by vertical dashed lines in each panel) using DiffMark strategy is noticeably greater than the alternative strategies.

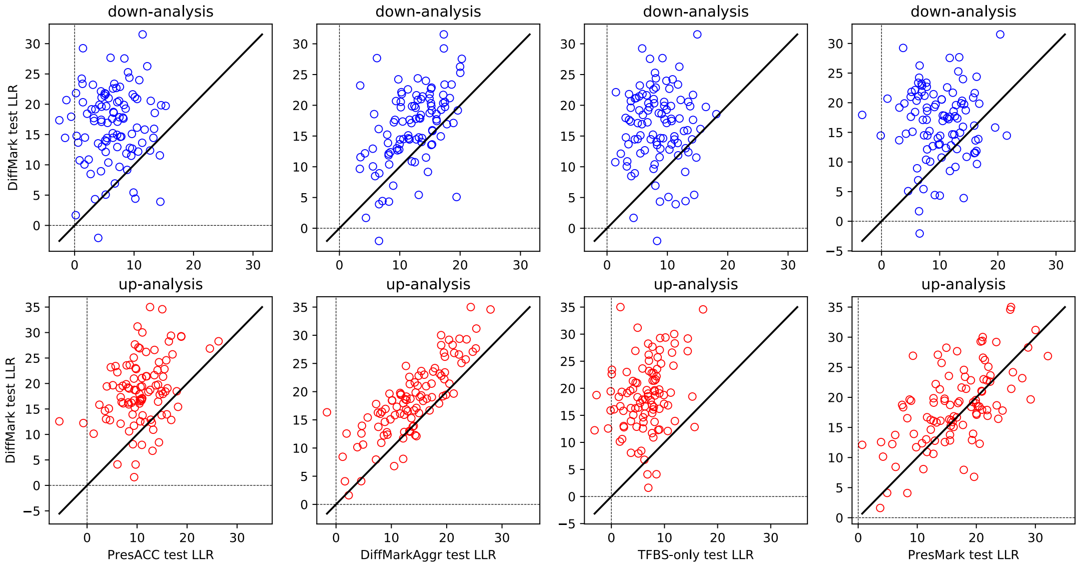

Figure S11. Head-to-head comparison of DiffMark strategy with alternative strategies. Test LLRs of the DiffMark strategy versus alternative strategies for up- and down-analyses, from 100 randomized repeats of training-validation-testing. Each point represents the LLR of the unseen (test) set of genes computed by the model trained with the optimal values of regularization coefficient and distance threshold.

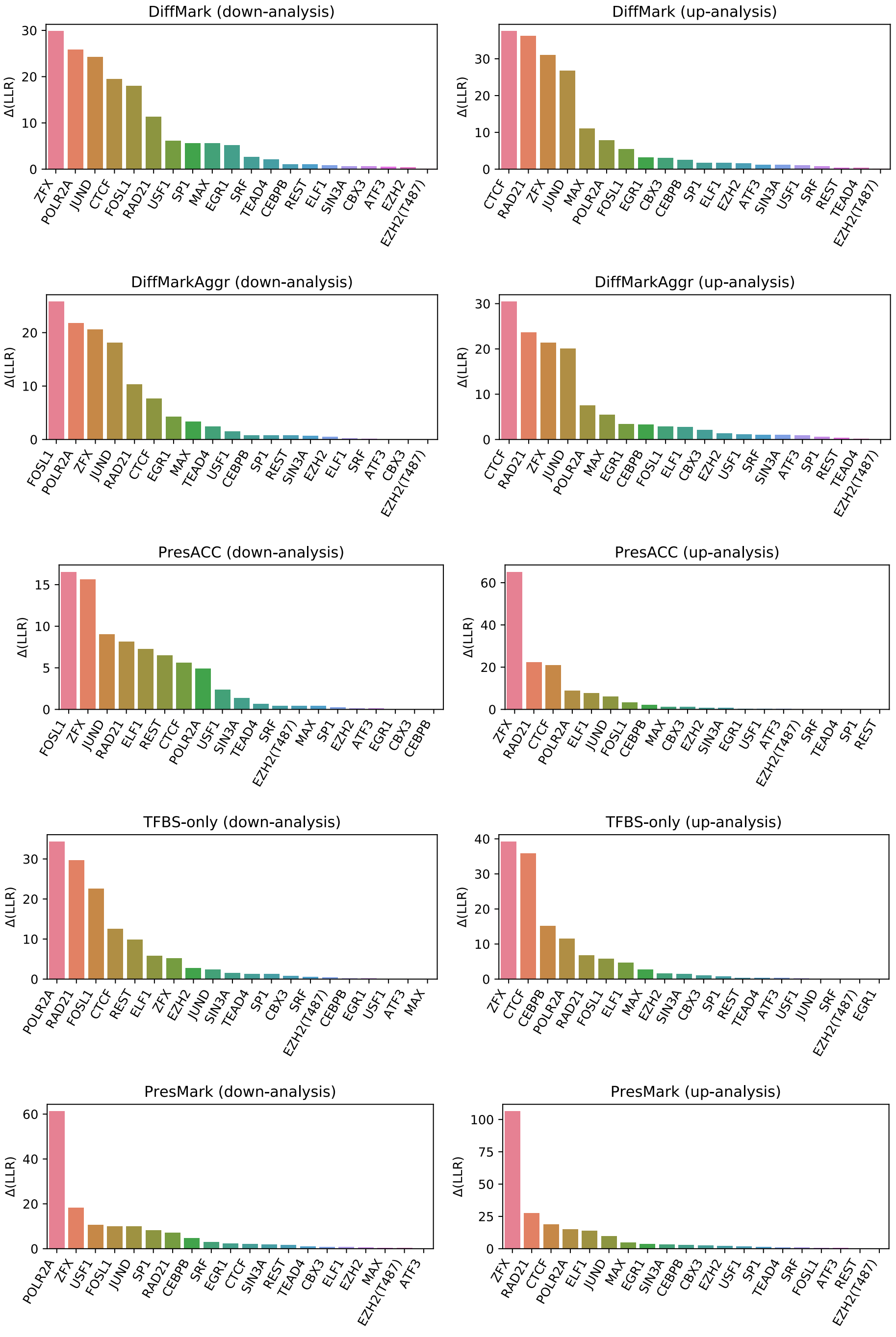

Figure S12. Model-based ranking of TFs, in down-analysis (left) and up-analysis (right), for each strategy. Model-based ranking of TFs, in down-analysis (left) and up-analysis (right), for each strategy. Note that the optimal regularization coefficients and distance threshold derived for each strategy (**Figure S4**) were used to retrain the model on the entire data set, in order to determine TF ranks by $\Delta(LLR)$. JUND, one of the key TFs predicted to be involved in CRC progression, is less prioritized in TFBS-only strategy compared to other strategies.

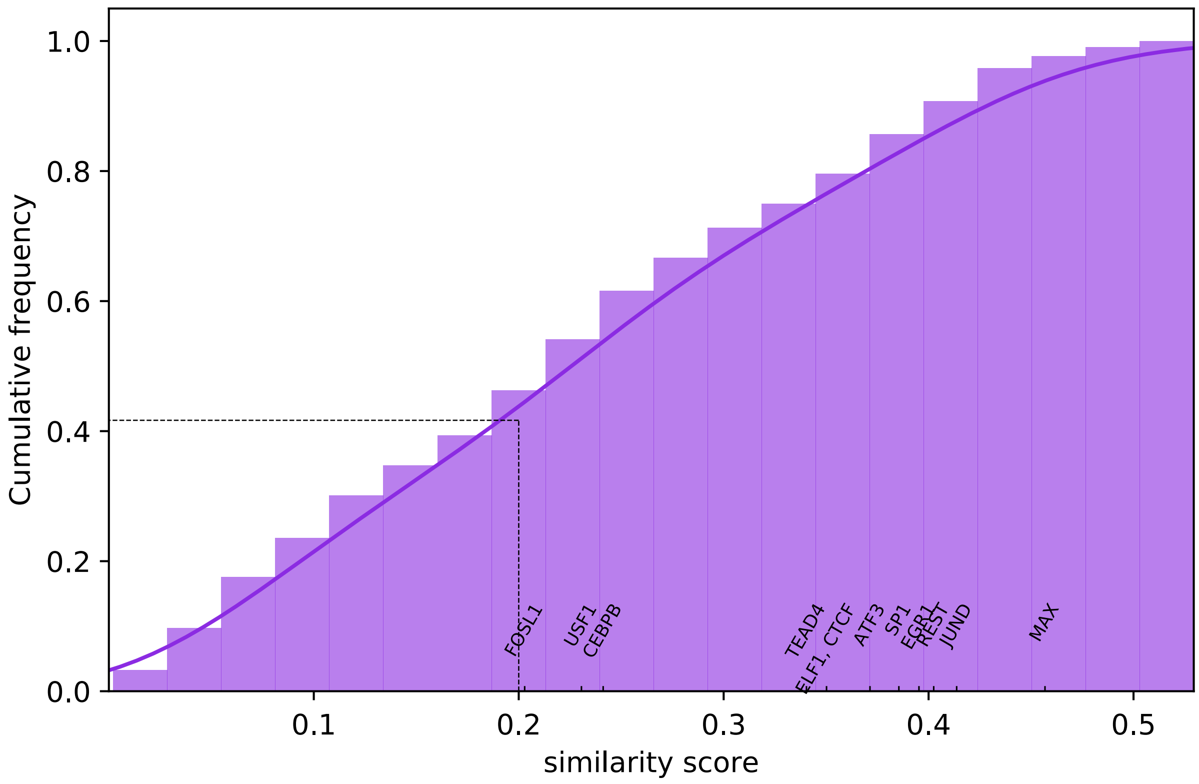

Figure S13. Cumulative frequency distribution of the maximum similarity of each K562 TF to a HCT116 TF. Cumulative frequency distribution of the maximum similarity of each K562 TF to a HCT116 TF. The similarity for each pair (K562 TF, HCT116 TF) was measured by the Jaccard similarity score between aggregated DiffMark cis evidence vector (each dimension is a gene), as described in text. For each K562 TF, the maximum similarity score to any HCT116 TF was recorded and contributes to this histogram. Based on this distribution, we noted the least score (~0.2) among those TFs that were present in the K562 set as well as the CRC set (marked on x-axis). This was used as the threshold to define the K562 TFs that are distinct from CRC TFs.

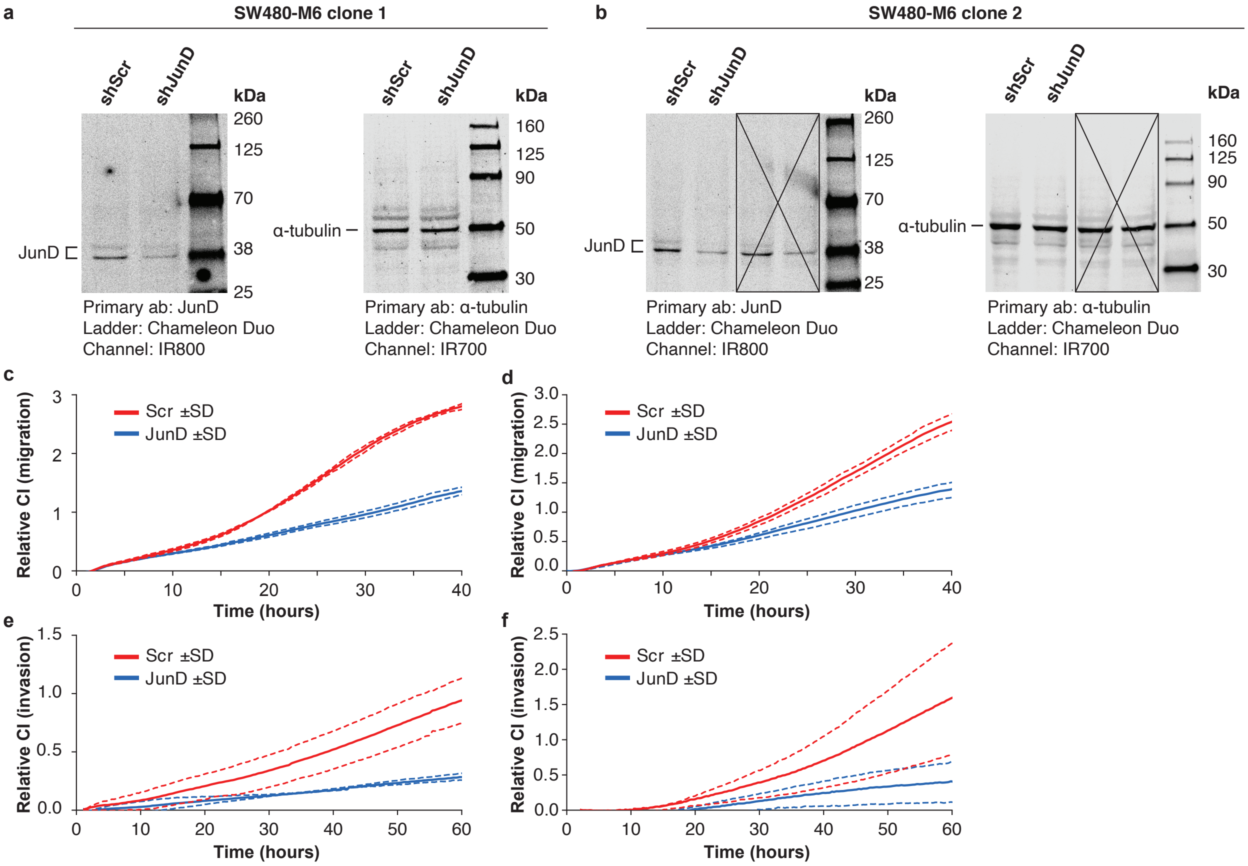

Figure S14. Knockdown of JunD impairs migration and invasion in invasive SW480-M6 cells. a,b. Immunoblot of JunD and α-tubulin in lysates from two independently generated SW480-M6 lines expressing JunD shRNA (shJunD) or scramble control (shScr). **c,d.** Migration for JunD knock-down (blue line) and scramble control (Scr; red line) was monitored continuously over 40 hours using a xCelligence realtime cell analysis platform with cell invasion migration (CIM) plates. Fetal bovine serum was used as a chemoattractant. Cell index (arbitrary units) corresponds to cell migration capacity. Dotted lines represent the standard deviation (SD) of three independent cultures measured in parallel. **e,f.** Cell invasiveness was measured continuously for 60 hours using CIM plates that were precoated with Matrigel. All other parameters are the same as for panels c-d. Panels a, c, and e contain data from one SW480-M6 cell line. Panels b, d, and f contain data from a second independently generated SW480-M6 cell line.

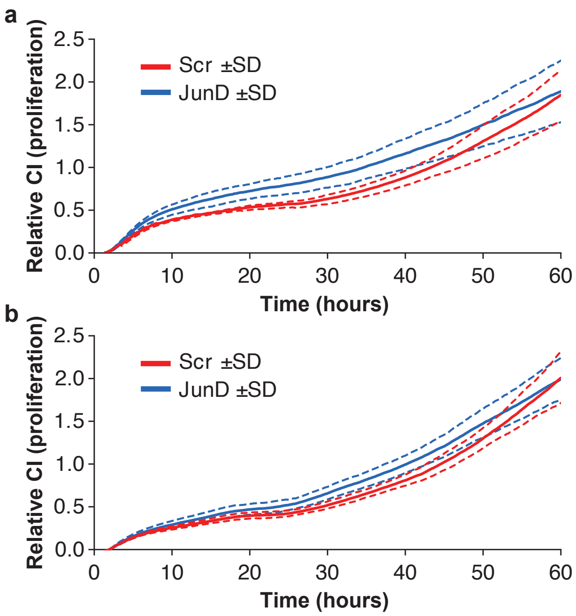

Figure S15. Cell proliferation is unaffected by JunD knockdown. Proliferation was measured for SW480 M0 **(a)** and M6 **(b)** cell lines following shRNA-mediated knockdown of JunD (JunD) or using a non-targeting control (Scr). Solid lines represent the mean of 3 independent wells assayed in parallel. Dashed lines represent the standard deviation.
